# Parametric Studies of the Load Transfer Platform Reinforcement Interaction with Columns

**DOI:** 10.3390/ma14144015

**Published:** 2021-07-18

**Authors:** Beata Gajewska, Marcin Gajewski, Zbigniew Lechowicz

**Affiliations:** 1 Department of Geotechnical Engineering, Institute of Civil Engineering, Warsaw University of Life Sciences, Nowoursynowska 159, 02 776 Warsaw, Poland; zbigniew_lechowicz@sggw.edu.pl; 2 Department of Strength of Materials and Theory of Elasticity and Plasticity, Institute of Building Engineering, Warsaw University of Technology, Al. Armii Ludowej 16, 00 637 Warsaw, Poland; m.gajewski@il.pw.edu.pl

**Keywords:** geosynthetics, LTP reinforcement, piled embankments, membranes

## Abstract

When designing embankments on a soft ground improved with columns (rigid inclusions) and with a geosynthetically reinforced load transfer platform (LTP), the methods of determining strains in reinforcement reduce the spatial problem to a two-dimensional one, and analytical calculations are carried out for reinforcement strips in the directions along and across the embankment. In addition, the two-dimensional FEM models do not allow for a complete analysis of the behavior of the reinforcement material. The aim of this research was to analyze the work of the membrane in the 3D space modeling of the LTP reinforcement, depending on the interaction with the column, the shape of the column’s cap, the value of the Poisson’s ratio, the value of the stiffness of the elastic foundation (subgrade reaction k) modeling of the soft soil resistance between the columns and the load distribution over membranes that model the reinforcement. The membranes were modeled in the framework of the theory of large deformations using the finite element method and slender shell elements as three-dimensional objects. This modeling method allowed for the analysis of the behavior of the LTP reinforcement in various directions. The conducted analyses showed, among others, that in the absence of soil resistance between the columns, regardless of the shape of the cap (square, circle), the greatest strains are located near the edge of the cap in the diagonal direction between the columns.

## 1. Introduction

**Figure 1:**
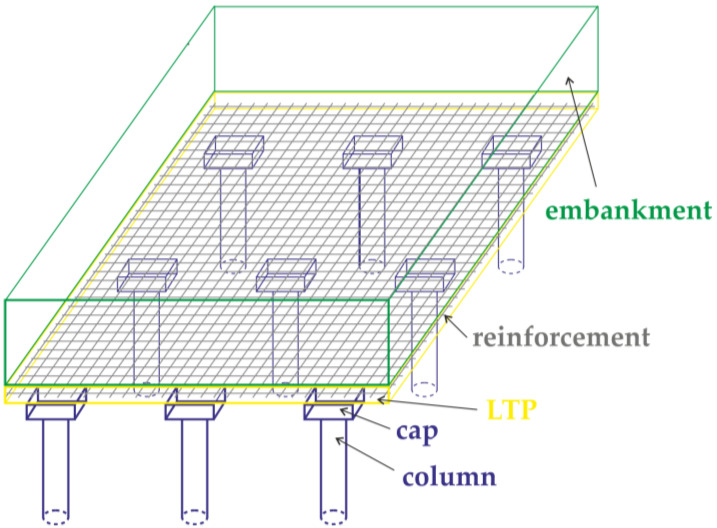
Basal reinforced piled embankment—arrangement of layers.

Currently, more and more roads are being built in areas with soft soils of considerable thickness. In this case, the ground needs to be improved. One of the methods for ground improvement is the use of rigid inclusions/columns (RI) made from properly prepared working platforms [1]. An embankment is built on improved ground. The embankment and traffic loads are transferred to the improved ground through the load transfer platform, which is usually reinforced (Figure 1). Woven geogrids are usually used as reinforcement, as well as woven; sometimes reinforcement from steel mesh is also used [2].

Building embankments on a soft soil improved with columns and with an LTP reinforced with geosynthetics is a very complex issue and requires an analysis of the interaction of individual system elements. In soil, an arching effect is created, first described by Terzaghi in 1943 [3], thanks to which a significant part of the load is transferred directly to the columns. The arching effect is enhanced by the use of LTP reinforcement [4]. In general, the problem of the embankment interaction with the LTP and the soft soil improved with columns should be formulated as a fully three-dimensional (3D) problem [5]. A few such analyses were presented in the literature [5,6,7,8,9], but they are very time-consuming. However, numerical analyses are often reduced to two dimensions by assuming plane strain conditions or axial symmetry when the behavior of one column is analyzed, e.g., [10,11,12,13,14,15,16,17]. Wijerathna and Liyanapathirana [18] compared numerical predictions from 2D-EA (two-dimensional model based on the equivalent area approach) and the 3D models and proposed a method to quantify the parameters for the EMC (an extension of the Mohr-Coulomb model) model simulating the column behavior when converting a 3D field embankment into a 2D-EA model or vice versa.

In the case of 2D analysis, deformations, forces, and bending moments in columns are determined using numerical methods (e.g., FEM). However, reducing the problem to a plane strain state means that a completely different system is analyzed. In this case, the columnar elements are modeled as walls of infinite length along the embankment. In order to analyze embankments on a soft ground improved with columns and with LTP reinforced with geosynthetics, it is necessary to properly calibrate the model, which requires specialist knowledge and experience. Numerical methods (especially 2D) often significantly underestimate the values of tensile forces in geosynthetic reinforcement [19]. Therefore, analytical methods are recommended for checking the reinforcement of the transmission layer itself. In analytical methods, the reinforcement is usually treated as an isotropic membrane, but the task of stretching the membrane (2D in the case of the theory of small deformations and 3D in the case of the theory of large deformations) is reduced to a one-dimensional problem. The purpose of this procedure is to simplify the task as much as possible, which must be solved analytically, e.g., by using the Taylor series expansion of the membrane deflection function, cf. [20].

For the correct design of the LTP reinforcement, it is necessary to determine the strain in the reinforcement, which must be smaller than the limit strain of the reinforcement for a given type of structure. In the analysis of embankments on a soft ground improved with rigid columns with a reinforced load transfer platform, the reinforcement calculations using analytical methods are performed in two steps. In the first step, the load is split between the part carried by the columns and the rest transferred to the geosynthetic and the soft soil between the columns. In the second step, the stresses and strains in the reinforcement are calculated.

The distribution of the load is different depending on the adopted model of the arching [21,22,23,24,25]. Two commonly used models are the arching model according to Zaeske [23] used in the EBGEO [19] and the model according to Van Eekelen—the concept of concentric arches (Concentric Arches model—CA model [24,26]) adopted in CUR226 [27].

In analytical methods, it is necessary to reduce the real 3D system to a 2D system, and in the sense of the variables sought, to a one-dimensional system [20]. The load transmitted to the reinforcement and the soft soil is collected and loads the membrane strip along and across the embankment, respectively [19,26,27,28,29,30]. Calculations are performed in two directions, for the spacing of columns along and across the embankment and for reinforcement along and across the embankment. The strip has a width equal to the equivalent width of the column (the circle is changed to an equivalent square) and the length equal to the spacing of the columns (the distance between equivalent columns).

Analytical methods allow to analyze the behavior of the LTP reinforcement only in the directions along and across the embankment [31]. To analyze the behavior of the reinforcement in different directions, it is necessary to use numerical methods. The aim of this article is to analyze the work of the isotropic LTP reinforcement modeled as a membrane depending on the way its interaction with the column is modeled, the shape of the column cap, the value of the Poisson’s ratio, and the value of the stiffness of the elastic foundation modeling the action from the soft soil under the reinforcement. In this work, membranes are modeled under the theory of large deformations using the finite element method and slender shell elements as three-dimensional objects. This procedure allowed for a better understanding of the LTP reinforcement working mechanisms. It should be expected that in the standard design formulation, where the work of reinforcement is assessed on the basis of a one-dimensional task, many of the phenomena are ignored.

## 2. Numerical Model of Reinforcement and Its Calibration

### 2.1. A Full Scale Experiment

**Figure 2:**
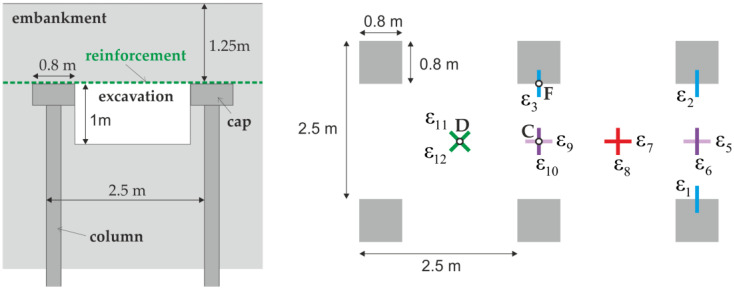
Full scale experimental setup according to [26,32,33]; C, D, F—points acc. Figure 3.

Almeida et al. [32] presented a full scale experiment. The plan of the test section is shown in Figure 2. In the analyzed experiment, the column spacing is 2.5 m. The columns are topped with 0.8 m wide square caps. In the analyzed area, before placing the geosynthetic reinforcement, a 1 m deep excavation was made. The lack of soil under the reinforcement means that the stiffness of the ground in this case is equal to zero. This models the most unfavorable situation—the loss of soil support, which may occur, for example, as a result of given inadequate consolidation of the soft soil layer, lateral excavation, or lowering of the groundwater table [19].

### 2.2. Results of Strains and Displacement Measurements of the Reinforcement

**Figure 3:**
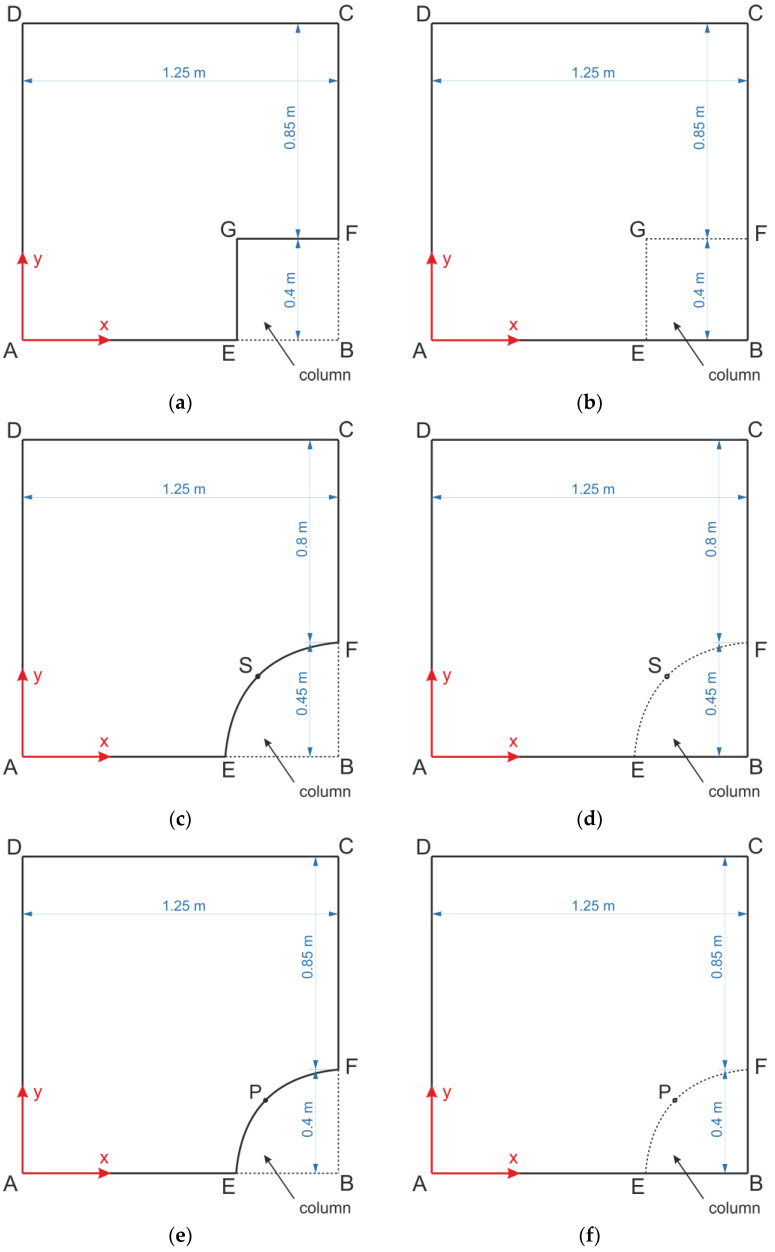
Geometric variants adopted in the FEM models of the reinforcement: (**a**) variant A1, (**b**) variant A2, (**c**) variant B1, (**d**) variant B2, (**e**) variant C1, (**f**) variant C2.

During the experiment presented in [32], the strains in the reinforcement as well as its vertical displacements were measured. The arrangement of the sensors is shown in Figure 2, and the results of the strain measurements are summarized in Table 1. The vertical displacements were measured between the column caps, in places corresponding to points C and D in Figure 3. At point C, the displacements equaled 0.17 m, and at point D 0.36 m.

### 2.3. FEM Numerical Model

The reinforcement at the base of the embankment was analyzed using the finite element method. The scheme of the task is shown in Figure 3. The ABAQUS/Standard [34,35] finite element method software was used to analyze the behavior of the reinforcement.

The reinforcement was modeled with finite elements of the shell type (S3 and S4R type elements) with negligible bending stiffness. The possibility of using this type of elements for the LTP reinforcement analysis was demonstrated in [36]. As the isotropy assumption is made, the isotropic and linear-elastic material are characterized by two parameters: Young’s modulus and Poisson’s ratio. The thickness of the layer modelling the reinforcement and the value of Young’s modulus were assumed in such a way as to obtain the effective stiffness as in Table 2 (reinforcement stiffness). As for the Poisson’s ratio, it is arbitrarily taken as 0.3 in many publications in this field. This work includes an analysis of its impact on the obtained results. In this research, the reinforcement was modeled as an isotropic membrane, which is a reference to analytical models. Consideration of the orthotropic properties of geosynthetics is the subject of further research. For some types of geogrids (hexagonal geogrids), the isotropic model may be sufficient due to the distribution of forces in the reinforcement [37]. The impact from the soft soil lying under the reinforcement was modeled using the classic one-parameter Winkler model (the material parameter here is the so-called subgrade reaction coefficient k). The Winkler constitutive model used for modelling the reaction of the soft soil assumes the linear relationship between the vertical force from membrane pressed into the soft soil and its vertical deflection (the proportionality coefficient is named as k). In the finite element formulation presented here, it means that in all nodes of the FEM mesh, the spring with effective stiffness highlights the reaction from the subsoil. In general, this model is only valid in a certain range, limited by plastic deformations in the subsoil.

The self-weight of the embankment is the only load considered. The traffic load was not considered as it was not included in the experiment. The load distribution on the membrane (modeling reinforcement) and columns was adopted analogically to that in [38]. The value of the load acting on the membrane between the columns was assumed according to the CA model [24] for the parameters as in Table 2. The problem of stretching the uniformly loaded shell/membrane model of the reinforcement in all cases was solved in the framework of the large deformation theory available in the ABAQUS software under the NLGEOM (Non-Linear GEOMetry) option. It is worth emphasizing that taking this option into account does not only lead to distinguishing the configuration of the deforming shell (the so-called non-linear geometry), but also to non-linearity in the constitutive relationship. Taking the NLGEOM option formally leads to the replacement of the strain tensor (as in the theory of small strains) by the logarithm of the left Cauchy-Green stretch tensor and the small deformation stress tensor with the Kirchhoff stress tensor [39,40]. Such a replacement of the strain and stress measures means that in the case of the limit transition to the theory of small displacements, the interpretation of material constants in relation to the classical Hooke’s relationship remains unchanged. The application of the theory of large deformations in the analyzed problem is a necessary condition, because even in the case of one-dimensional membrane tensile problems used in the analytical formulation [19,27], the deformations are calculated in relation to the current configuration and not the initial one (as a value related to the local arc length).

The application of such finite element formulation (within large deformation theory) is needed for reasonable physical modelling of the analyzed problem. Usage of the small deformation theory leads to ambiguous displacement fields in which vertical displacement drastically changes when crossing the boundary between columns and soft soil infill. The other reason for such choice is the fact that even in the standard designing approach (the analytically formulated problem of uniaxially extended membrane) for evaluation of the average strain in the reinforcement, the large deformation is used. This fact leads to the well-known problem of obtaining the solution in the non-closed form and that is why, for example in EBGEO [19], the nomograms are used for determination of the forces in membrane modelling reinforcement.

In order to evaluate the interaction between the reinforcement and the column, six variants, namely A1, A2, B1, B2, C1, and C2, respectively, were adopted for the analysis; see Figure 3. There is a difference between the variants A and B (or C) in the shape of the column cap, while in the case of B and C, the shape does not change (it is circular), but the radius changes so that it is possible to also take into consideration the so-called effective radius of the column cap. The adoption of the round shape of caps for parametric analysis results from the typical shape of the columns. In variants B1 and B2, the size of the caps was assumed to be equivalent to the size of the square caps (equal areas). On the other hand, in variants C1 and C2, the column diameter was assumed to be equal to the width of the square cap to ensure the same clear distance between the columns in the directions along and across the embankment. The difference between the variant A1 and A2 (and B1 and B2 (or C1 and C2, respectively) is the method of taking into account the interaction of the reinforcement with the surface of the column cap. In the first case, zero displacement boundary conditions at the edge of the cap were assumed, and in the second, the continuity of the reinforcement over the columns was modeled, taking into account the fact that reinforcement is resting on the column with a stiffness many times greater than that of the soft soil between the columns. The columns were modeled with the Winkler model with a stiffness equivalent to that of a 12 m long concrete column (k_c_ = 2.15 × 10^10^ kN/m^3^).

In the cases modeling the experiment (A1 and A2), there is no reaction from soft soil, so in this case, k = 0 is assumed. In the parametric analysis, *k* values ranging from 0 to 500 kN/m^3^ were adopted in order to assess the influence of soil resistance on the behavior of the membrane.

Going into detail, e.g., in the case of the variant A1, at the boundaries EG and GF, zero displacement boundary conditions in all three directions (u_x_ = u_y_ = u_z_ = 0) are assumed. The same was done for variants B1 and C1 on the edge EF. In addition, a symmetry with respect to the x axis was assumed on the AE and DC boundaries, and a symmetry with respect to the y axis on the boundaries AD and CF.

In case of boundary conditions u_z_ = 0 on the column edge, we have the same situation like in case of the analytical solution. Having this result allows us to compare the solutions with the designed standard one. Of course, it could be assumed that there is the Winkler relationship for nodes on the columns edges in the form R_z_ = k * u_z_ instead, but then the deflection function would have exactly the same shape only shifted down. In addition, the strain functions would be the same, and that is the critical designing parameter.

In the case of the A2 variant, the symmetry with respect to the x axis was assumed on the edges AB and DC, and symmetry with respect to the y axis on the boundaries AD and BC. The vertical movement of the modeled reinforcement is blocked by Winkler’s reaction forces in the column and subsoil regions.

In the case of the other variants, the respective displacement boundary conditions are implemented in an analogous manner.

The part of the load from the embankment transferred by the reinforcement was adopted in accordance with the concept of load distribution according to CA [24,26] on the reinforcement surface. The parameters used for the analysis are listed in Table 2.

### 2.4. Convergence Analysis

In order to assess the influence of the FEM mesh quality on the obtained results, the convergence was assessed for two cases—B1 and B2. The obtained conclusions can be safely transferred to other variants. Of course, in the case of the variants A1 and A2, at point G, significant concentrations of stresses and strains is expected, but this is natural in the case of the so-called “sharp corner”. The standard procedure is to exclude the so-called singularity points from the convergence analysis.

**Figure 4:**
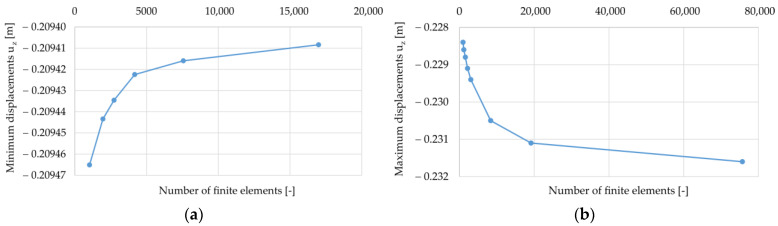
Minimum vertical displacements u_z_ at half the distance between columns depending on the number of elements: (**a**) variant B1; (**b**) variant B2.

**Figure 5:**
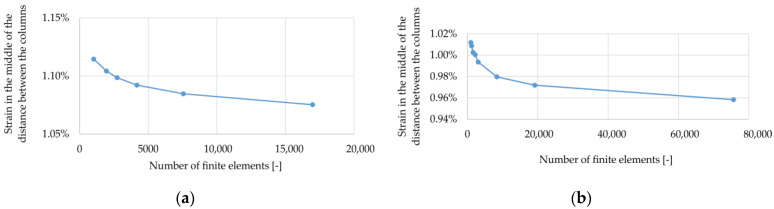
Strains at point C (see Figure 2) depending on the number of elements: (**a**) variant B1; (**b**) variant B2.

**Figure 6:**
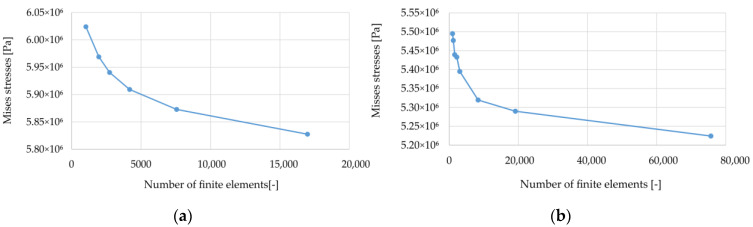
Mises stresses at point C (see Figure 2) depending on the number of elements: (**a**) variant B1; (**b**) variant B2.

For both cases, the influence of the number of finite elements on the minimum values of vertical displacements (they have a negative value in the adopted coordinate system) of the reinforcement u_z_, and the values of strains and Mises equivalent stresses at point C (half the span between the columns) were analyzed (see Figure 3). The obtained results are presented in the graphs shown in Figure 4, Figure 5 and Figure 6.

**Figure 7:**
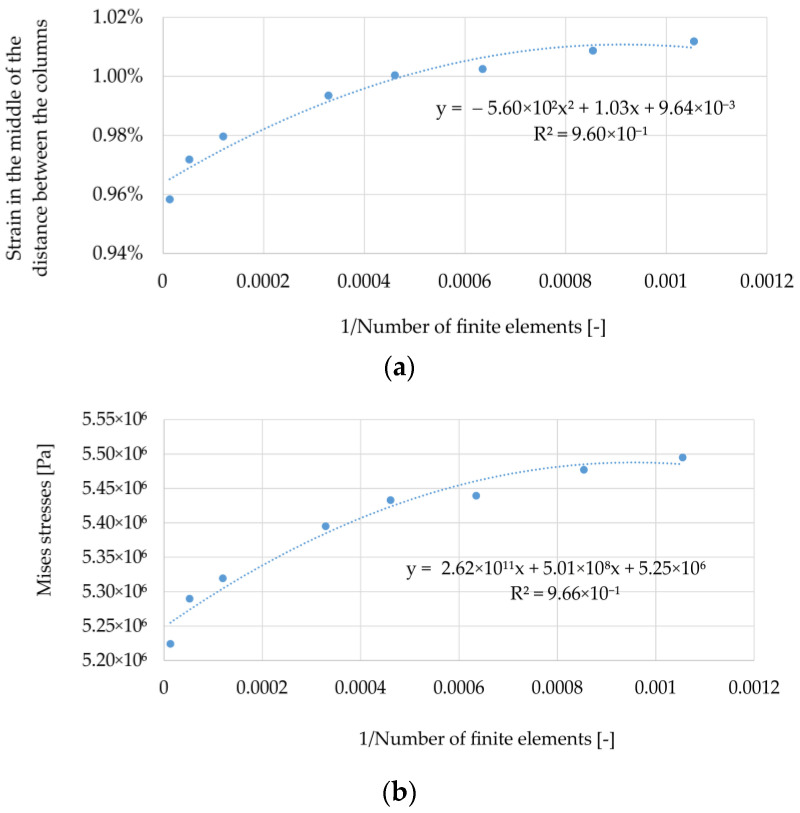

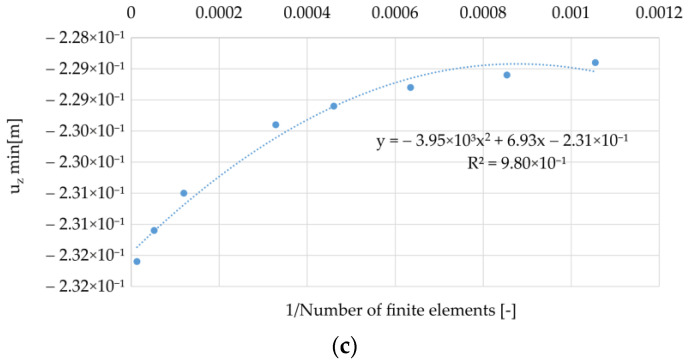
(**a**) Strain in the middle between columns; (**b**) Mises stresses, (**c**) u_z_ min, as a function of 1/N—approximation.

Analyzing the results presented in Figure 4, Figure 5 and Figure 6, it can be noticed that with an increase in the number of elements, the solutions stabilize, but none of the cases obtained a situation where the line connecting successive points representing the solutions sought is inclined at an angle close to zero to the horizontal axis. Therefore, a decision was made to define the accuracy of the obtained solutions in relation to the estimate of the exact solution. For this purpose, graphs of the sought quantities as a function of the reciprocal of the number of elements were prepared; see Figure 7 (the procedure is presented on the example of the variant B2). In this axes system for 1/N going to zero, the solution tends to an exact solution (as N goes to infinity). Then, the results presented in this way were approximated by a quadratic function, obtaining coefficients of determination at a level above 0.96. Using the obtained approximation functions, their values at zero were determined, which in the further evaluation of the correctness of the mapping were used as reference solutions. The same was done in the case of the results obtained for the variant B1, but they are not presented in the form of graphs.

**Figure 8:**
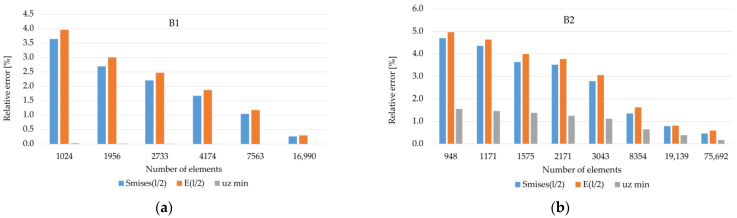
Relative error of individual quantities in relation to the approximated “reference” solution: (**a**) variant B1; (**b**) variant B2.

On the basis of the reference values obtained in this way, the relative error for meshes with a certain number of elements was determined and the results are presented in the form of a column chart in Figure 8.

Analyzing the above graphs, it can be concluded that for the B1 case, starting from about 2000 elements, the number of finite elements does not affect the values of the maximum deflection u_z_ of the reinforcement. Based on the graphs shown in Figure 8a, it was assumed that for the B1 case (and A1 and C1, respectively), the accuracy of calculations for approx. 7500 finite elements is satisfactory. On the other hand, for the case of B2 (and A2 and C2, respectively) (see Figure 8b), the results were obtained with a satisfactory accuracy for approx. 19,000 finite elements. Further increasing the number of elements significantly extended the computation time and generated problems with convergence for some of the analyzed variants of the parametric analysis.

The final number of finite elements for individual variants is presented in Table 3. In all cases, the shell S3 and S4R element with linear shape functions were used.

**Figure 9:**
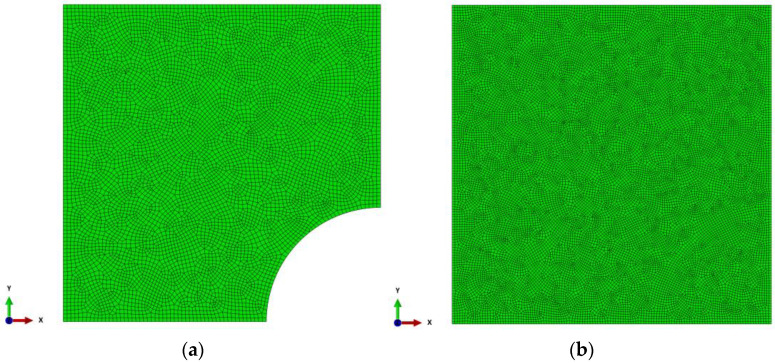
Meshes built from finite elements type S3 and S4R: (**a**) for the B1 case—7708 finite elements; (**b**) for case B2—19,139 finite elements.

Figure 9 shows examples of the finite element method relatively uniform meshes for variants B1 and B2.

### 2.5. Calibration of the Model with the Results of Deformation Measurements

**Figure 10:**
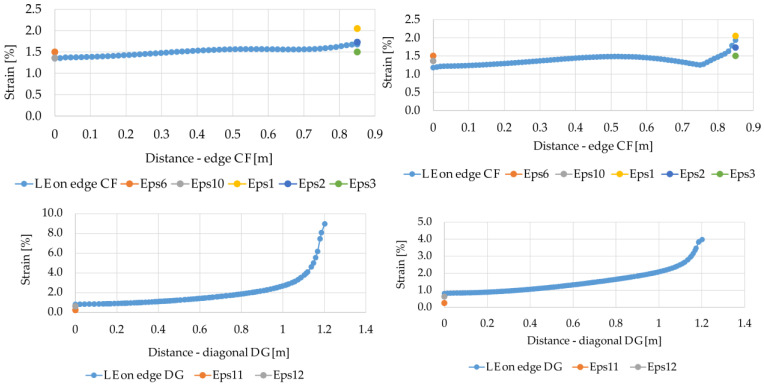

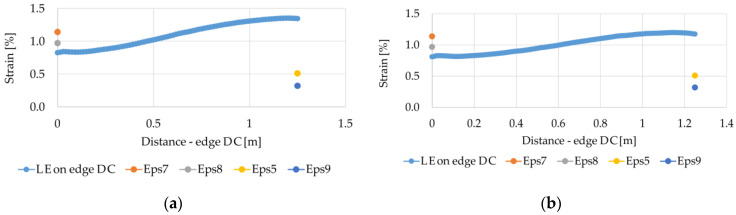
Comparison of the FEM calculation results with the results of strain measurements: (**a**) variant A1; (**b**) variant A2.

The numerical model (variant A1, see Section 2.3) was calibrated by comparing the results of the strain measurements with the results obtained using the finite element method. Figure 10a shows a graph of strain in the membrane for the variant A1 on the CF edge, corresponding to half of the distance between the columns along the y axis, on the diagonal DG, i.e., for the half of the largest distance between the columns and on the DC edge, i.e., between the point at the intersection of the diagonals between the four columns, and a point halfway between the columns along the y axis. The obtained results were compared with the values of the strain measurements obtained in the middle between the columns (point C) and on the edge of the column (point F). In the analyzed case A, the strains at point C (analyzed CF edge) were 1.34%, which is slightly less than the smallest result of the strain measurement at this point (1.36%). In turn, at point F, the strains obtained as a result of numerical analyses are slightly lower than the average value obtained from the measurements.

As can be seen in the area of the G point (diagonal DG), i.e., at the corner of the square cap, there is a sharp increase in the strains in the membrane. The corner is the point where the stress concentration occurs. Unfortunately, there is no data on strain measurements at this point, and therefore no reference point to verify to what extent the strain results obtained at point F reflect the actual behavior of the reinforcement in the structure. On the other hand, at point D, the strain was 0.83%, i.e., more than the results from the measurements.

When analyzing the results of strains at the DC edge, it can be stated that in the case of point D, the results were lower than the measurement results, and in the case of point C, significantly greater than the measurement results. The measurement results for $\varepsilon_{5}$ and $\varepsilon_{9}$ are surprisingly low. It was also pointed out by Van Eekelen [26] and it also applies to the measurements for $\varepsilon_{11}$ and $\varepsilon_{12}$.

Additionally, the results of strains determined according to the variant A2 were compared at individual points with the measurements (Figure 10b). In this case, a slightly lower compliance of the calculations with the measurements at point C was obtained, while in the case of the variant A2, the increase in strains at point G is much smaller compared to the variant A1.

## 3. Research Results and Discussion

### 3.1. Effect of the Geometric Model on the Obtained Results

**Figure 11:**
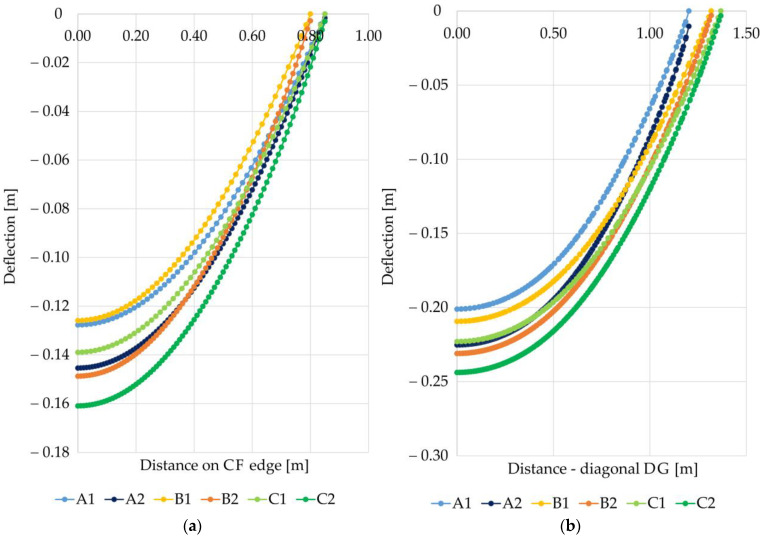
Deflection of the membrane modeling reinforcement depending on the adopted variant; reinforcement stiffness J = 1615 [kN/m]: (**a**) on the edge CF (between columns); (**b**) on the diagonal DG.

Analyzing the deflection curves at the CF edge and diagonal DG (Figure 11) of the membrane modeling the reinforcement of the load transfer platform with a stiffness of 1615 kN/m depending on the variant used, it can be seen that greater maximum deflection is obtained in cases with zero displacement conditions at the edge of the column (variants A1, B1, C1). At the edge DG, the greatest deflection was obtained for the variant C2, and the smallest for the variant B1. The lowest deflection value between the columns was obtained for variant B1. The difference between the smallest deflection between the columns for the variant B1 and the maximum obtained value for the variant C2 in this case is 27.85%. On the other hand, the difference between the minimum value of deflection at the intersection of diagonals between the columns obtained for the variant A1 and the maximum value obtained for variant C2 is 21.26%. The deflection curves at the CF edge of the membrane modeling the reinforcement of LTP with a stiffness of 1615 kN/m depending on the considered variant are shown in Figure 11b. At the intersection of the diagonals between the columns, there are greater deflections of the reinforcement than in the middle between the columns in the x and y directions.

**Figure 12:**
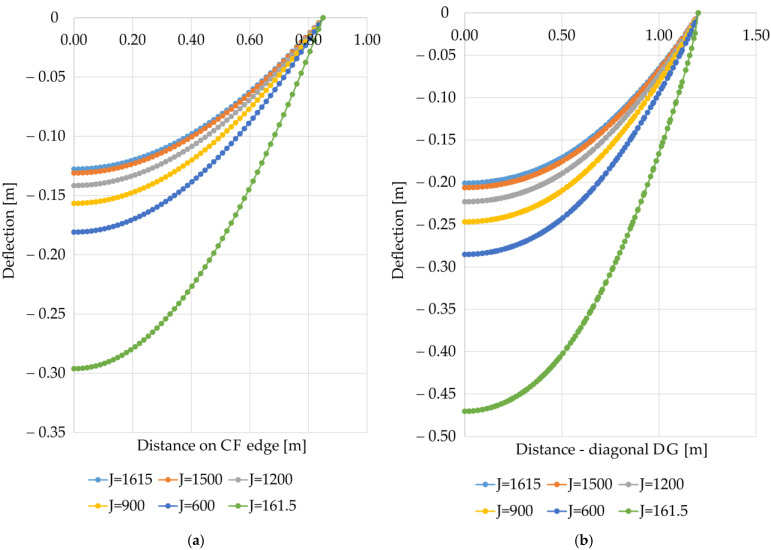
Deflection of the membrane modeling reinforcement for the variant A1 depending on the stiffness of the reinforcement: (**a**) on the edge CF (between columns); (**b**) on the diagonal DG.

Figure 12a shows diagrams of the membrane deflection at the CF edge depending on the reinforcement stiffness for the variant A1. The maximum deflection in the middle of the span between the columns increases with the decrease in the stiffness of the reinforcement, with a 10-fold change in stiffness resulting in a 2.32-fold increase in the value of the maximum deflection. A similar relationship was observed in the case of the analysis of the reinforcement behavior on the diagonal DG. In the case of the variant A1, the 10-fold change in stiffness caused a 2.34-fold increase in the value of the maximum deflection of the reinforcement at the diagonal intersection between the columns (see Figure 12b). From a practical point of view, further experimental and numerical analyses should be carried out in order to assess the influence of different longitudinal and transverse stiffness of the reinforcement on the behavior of a geosynthetically reinforced load transfer platform on a soft ground improved with columns.

**Figure 13:**
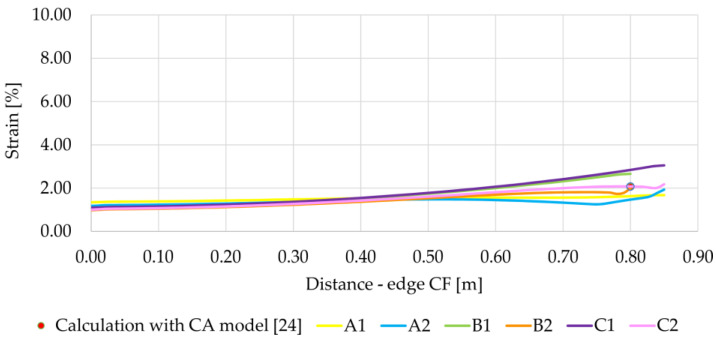
Reinforcement strain at the edge CF depending on the analyzed variant, J = 1615 kN/m.

The differences between the strain values at the CF edge for individual variants are greater at the edge of the cap (column) than in the middle between the columns (Figure 13). In the modeled experiment, the caps were square. In the case of the variants A1 and A2 (square caps, different boundary conditions), B2, and C2 (round cap, respectively, with a diameter equivalent to the square cap and with a diameter equal to the square cap (the same distance between the columns for square and round caps)), similar deformation values at the edge of the column were obtained. On the other hand, in the case of variants B1 and C1 (round cap, with a diameter equivalent to the size of the square cap, and with a diameter equal to the size of the square cap, respectively—zero displacement boundary conditions at the edge of the column/cap), the obtained deformation values at the edge of the column are significantly greater. In the case of variants A2, B2, and C2, the strains initially decrease on the edge CF towards the center between the columns, then rise and again decrease towards the point C (in the middle of the span between the columns). The strain in the membrane between the columns along the CF axis is the most uniform in the case of the variant A1. Pham and Dias [4] showed that the calculations of the CUR226 CA analytical model [24,27] allows to obtain the results of reinforcement strain closest to the obtained measurements. The performed calculations were compared to the results obtained according to the CA model for the data given in Table 2 (Figure 13). The highest compatibility between the analytical calculations and the numerical model was obtained for variants B2 and C2.

**Figure 14:**
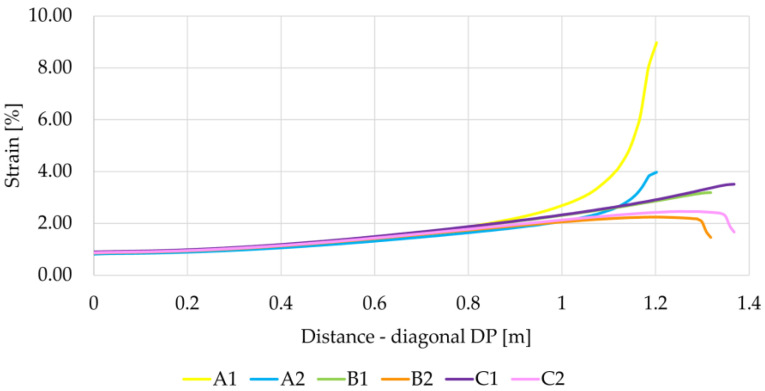
Reinforcement strain on the diagonal DP depending on the analyzed variant.

When analyzing graphs of membrane strains along diagonal DP (Figure 14), a much bigger difference in the value of strain at the edge of the column can be seen, depending on the geometric model adopted. In this case, the highest strains at the edge of the column were obtained for the variants A1 and A2, while the strains for the variant A1 are over six times greater than the strains for the variant B2, for which the strains at the edge of the column are the smallest.

In conclusion, it can be stated that the shape of the cap has a great influence on the strains of the geosynthetic reinforcement. The significant effect on the maximum strain in the reinforcement was also reported by Zhang et al. [38]. However, the case when the subgrade reaction of soft soil is assumed as 0 was not analyzed there.

**Figure 15:**
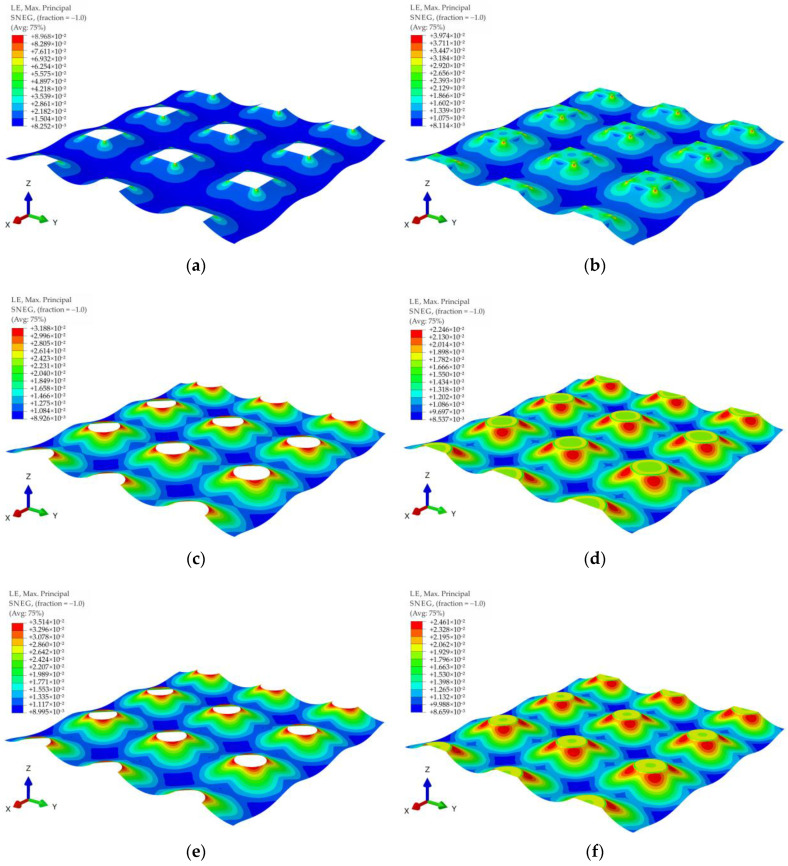
Membrane deformation and maximum principal strains for J = 1615 kN/m and v=0.3: (**a**) variant A1; (**b**) variant A2; (**c**) variant B1; (**d**) variant B2; (**e**) variant C1; (**f**) variant C2.

Figure 15 shows contour graphs of reinforcement deformations for membrane stiffness J = 1615 kN/m and subgrade reaction of soil between columns k = 0 kN/m^3^ for the variants A1, A2, B1, B2, C1, and C2. The biggest strains in the case of the variants A1 and A2 are located near the corners of the column, in the diagonal direction between the columns (the largest distance between the columns). Interestingly, also in the case of a round cap, the greatest deformations are located near the edge of the column in the direction of the diagonal between the columns (see Figure 15c–f). However, this phenomenon occurs only in the lack or small values of the soft soil resistance (zero or low values of subgrade reaction coefficient).

### 3.2. Effect of the Poisson’s Ratio Value

When modeling the reinforcement material of the Load Transfer Platform using the Finite Element Method, the Poisson’s ratio value is usually assumed to be 0.3 [5,38]. For all of the variants A1, A2, B1, B2, C1, and C2, for the reinforcement stiffness, J = 1615 [kN/m] and the subgrade reaction of soft soil between the columns k = 0 [kN/m^3^], the influence of the Poisson’s ratio on the deformation values both between the columns (edge CF) and on the diagonal between the columns (GP direction).

**Figure 16:**
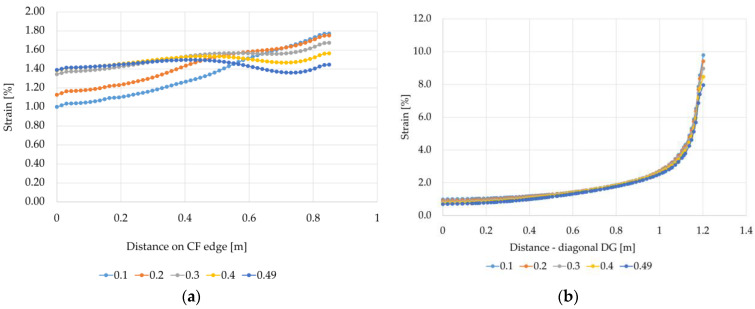
Graphs of membrane strains depending on the Poisson’s ratio value—variant A1: (**a**) the edge CF, (**b**) the diagonal DG.

When analyzing the strain in the reinforcement shown in Figure 16a, it can be seen that the value of the Poisson’s ratio has a significant impact on the values of the obtained results. The smaller the value of the Poisson’s ratio, the greater the difference between the strain of the reinforcement at the edge of the column and the strain in the middle between the columns. For variant A1, in the case of Poisson’s ratio equal to 0.3, the smallest strain is 20.2% lower than the highest value obtained at the edge of the column. On the other hand, for Poisson’s ratio of 0.49, the difference between the strain between the columns and the strain at the edge of the column in relation to the strain at the edge of the column is only 4.1% smaller. In this case, the highest strain values were obtained at a distance of about 0.42 m from the center between the columns and they are 3.4% greater than the strain at the edge of the column. In turn, the smallest strain values were obtained at a distance of approx. 0.73 m from the center between the columns; they are 6.2% smaller than the strain of the reinforcement at the edge of the column. On the diagonal between the columns (diagonal DG), the strain values are the smallest in the middle at the intersection of the diagonals corresponding to point D (Figure 16b). The strain of the reinforcement at the corner in the analyzed case, depending on the value of the Poisson’s ratio, is from 9.9 (for v=0.1) to 11.5 (for v=0.49) times greater than the smallest strain values at the intersection of the diagonals. Such a large increase in deformation in the area of the cap corner is due to the concentrations of stresses in this area. The influence of the shape of the column/cap on the obtained strain values was also noted in [33,38,41].

**Figure 17:**
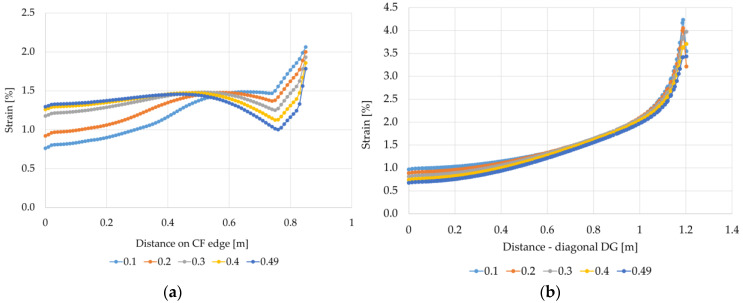
Charts of membrane strains depending on the Poisson’s ratio value—variant A2: (a) the edge CF, (**b**) the diagonal DG.

Analyzing the strain graphs at the edge CF for the variant A2 (Figure 17a) and for different values of the Poisson’s ratio, a different character of the membrane behavior in the column area compared to the variant A1 can be seen. The strain grow in the vicinity of the column and at the edge of the column are from approx. 14% to almost 23% greater than the strain according to variant A1. In turn, the strains in the middle between the columns are from 6.5% to 24% smaller than the strain for the variant A1. Reinforcement strains determined according to the variant A2 at the intersection of diagonals are at a similar level as calculated according to the variant A1. On the other hand, the strains at the column (the corner of the cap) are much smaller. Interestingly, in this case for v=0.1 and v=0.2, the membrane strain significantly decreases just next to the corner of the cap (Figure 17b). It could be caused by membranes wrinkling in the area.

**Figure 18:**
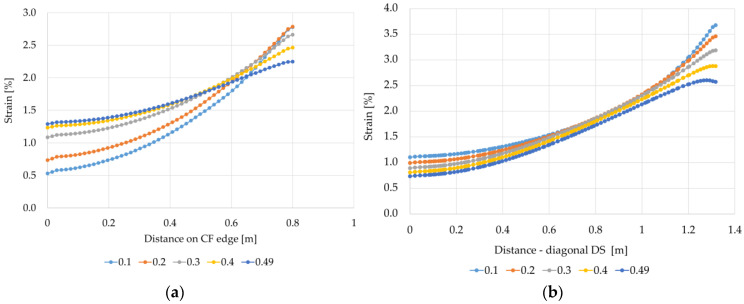
Graphs of membrane strains depending on the Poisson’s ratio value—variant B1: (**a**) on the edge CF; (**b**) on the diagonal DS.

**Figure 19:**
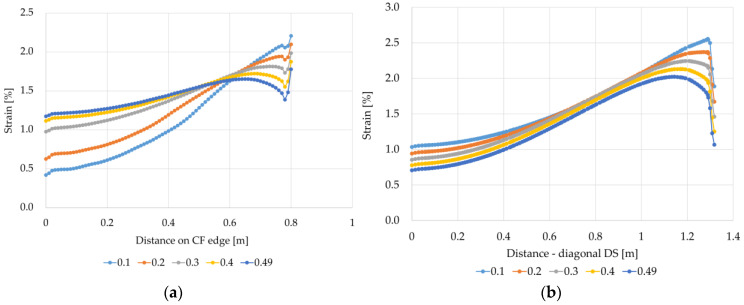
Graphs of membrane strains depending on the Poisson’s ratio value—variant B2: (**a**) on the edge CF; (**b**) on the diagonal DS.

In the case of variants B1 and B2, a significant impact of the Poisson’s ratio values on the strain results can also be seen (Figure 18 and Figure 19). For the variant B1, as the Poisson’s ratio value increases, the difference between the maximum strain at the edge of the column/cap and the smallest strain in the middle between the columns (CF edge) decreases. For v=0.1, the strain at the column is 5.25 times higher than the strain in the middle of the span between the columns (point C). In turn, for v=0.49, the strain at the column is higher than the strain between the columns only 1.74 times. For the variant B2 (Figure 19), the behavior of the membrane modeling the reinforcement material near the cap/column is different from the behavior of the membrane for the variant B1 (Figure 18). At the edge CF, the highest strains occur at the edge of the column, then they decrease significantly, and further towards the center between the columns; they initially increase and further decrease again. The strain increase is bigger and the difference between the largest and smallest strains is smaller for higher values of the Poisson’s ratio. On the other hand, the strains just at the edge of the cap in the direction of the diagonal between the columns are much smaller than the largest ones just next to the edge of the cap, and as the Poisson’s ratio increases, the highest strain on the diagonal DS locate farther from the edge of the cap.

Summing up, due to the large impact of the Poisson’s ratio on the obtained results, its value for the reinforcement material should not be selected arbitrarily. The authors are aware that it would be very difficult to determine an adequate value of the Poisson’s ratio. Therefore, the selection of a specific value for calculations should be preceded by a thorough analysis.

### 3.3. Effect of Soft Soil Stiffness

**Figure 20:**
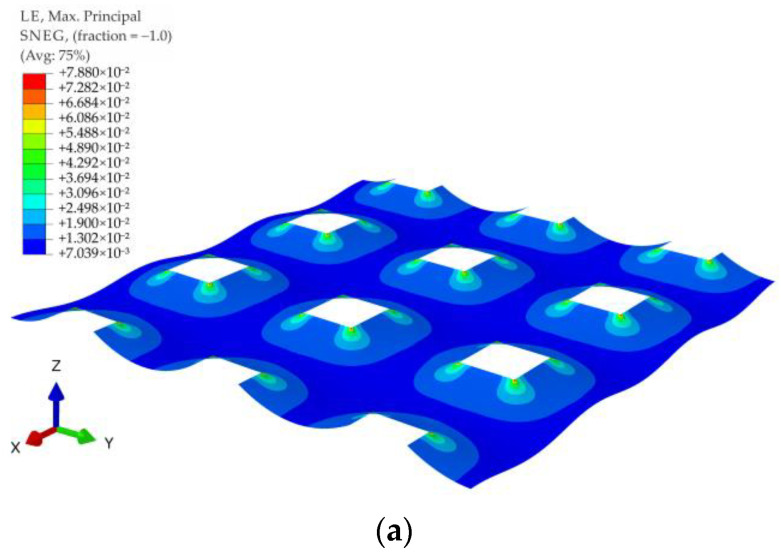

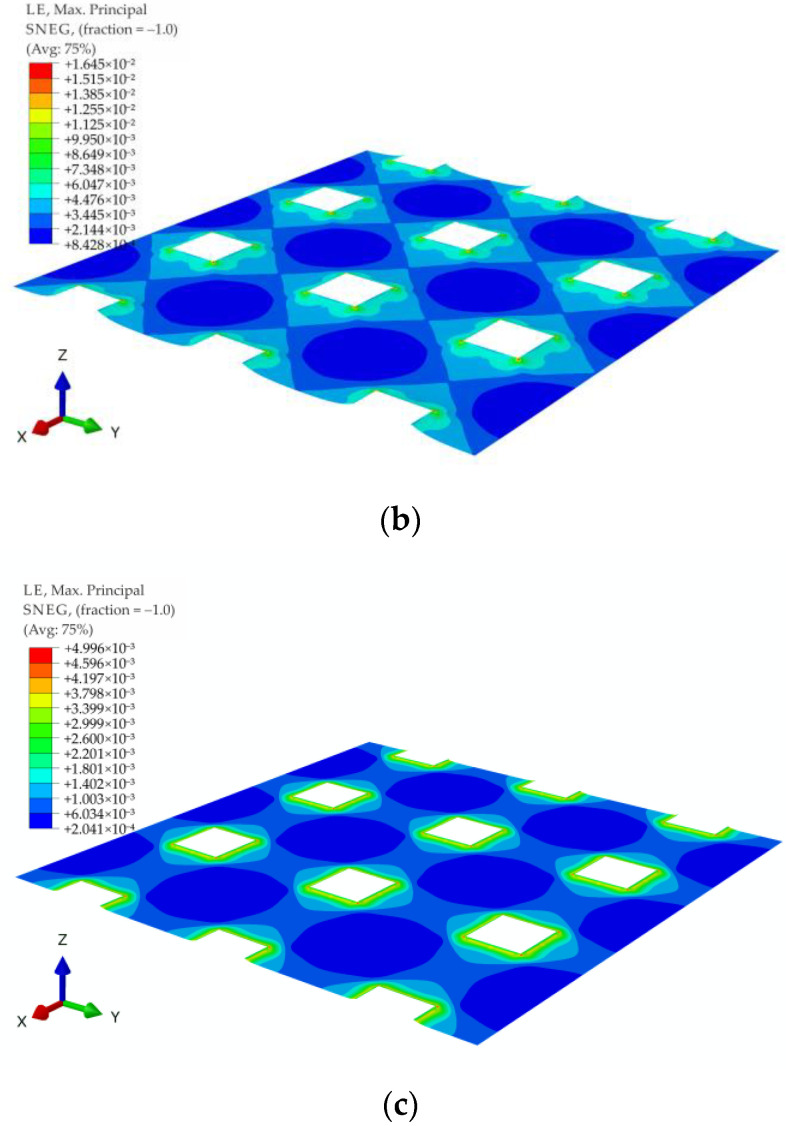
Deformation and maximum principal strain in reinforcement, variant A1: (**a**) k = 10 kN/m^3^; (**b**) k = 150 kN/m^3^; (**c**) k = 500 kN/m^3^.

**Figure 21:**
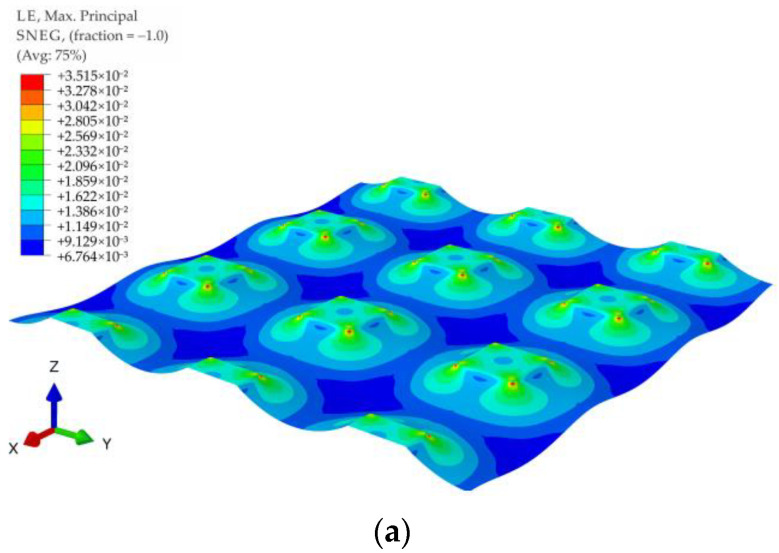

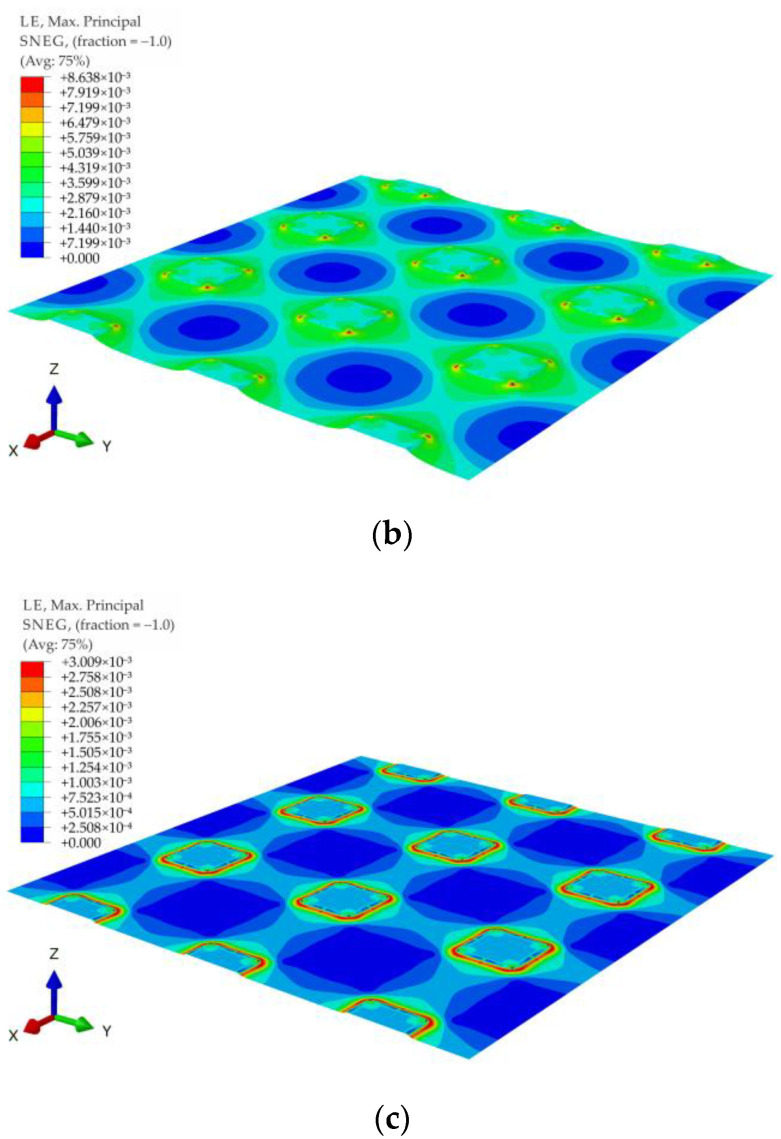
Deformation and maximum principal strain in reinforcement, variant A2: (**a**) k = 10 kN/m^3^, (**b**) k = 150 kN/m^3^, (**c**) k = 500 kN/m^3^.

For all of the geometric variants, i.e., A1, A2, B1, B2, C1, and C2 (see Figure 3), the influence of the subgrade reaction coefficient k modeling soft soil resistance on the behavior of the reinforcement above the columns was analyzed. The calculations were made for the subgrade reaction values k = 10 kN/m^3^, k = 50 kN/m^3^, k = 100 kN/m^3^, k = 150 kN/m^3^, k = 200 kN/m^3^, k = 300 kN/m^3^, k = 400 kN/m^3^, and k = 500 kN/m^3^. Figure 20 shows the distribution of maximum principal strains in the reinforcement modeling membrane according to the variant A1 for selected values of the subgrade reaction, respectively, k = 10 kN/m^3^, k = 150 kN/m^3^, k = 500 kN/m^3^, and in Figure 21 for the membrane according to variant A2 and the same subgrade reaction values. In the case of the variant A1, the highest strain of the reinforcement are located in the corners of the column/cap for all analyzed J/k ratios (the unit is m^2^). For J/k = 3.23 (k = 500 kN/m^3^), there is a cumulation of equal size strains around the column, with the cumulation of the highest strains at the corners still clearly visible. In turn, for the variant A2 for the ratio J/k = 3.23, the highest strain of the membrane distribute uniformly around the column/cap.

**Figure 22:**
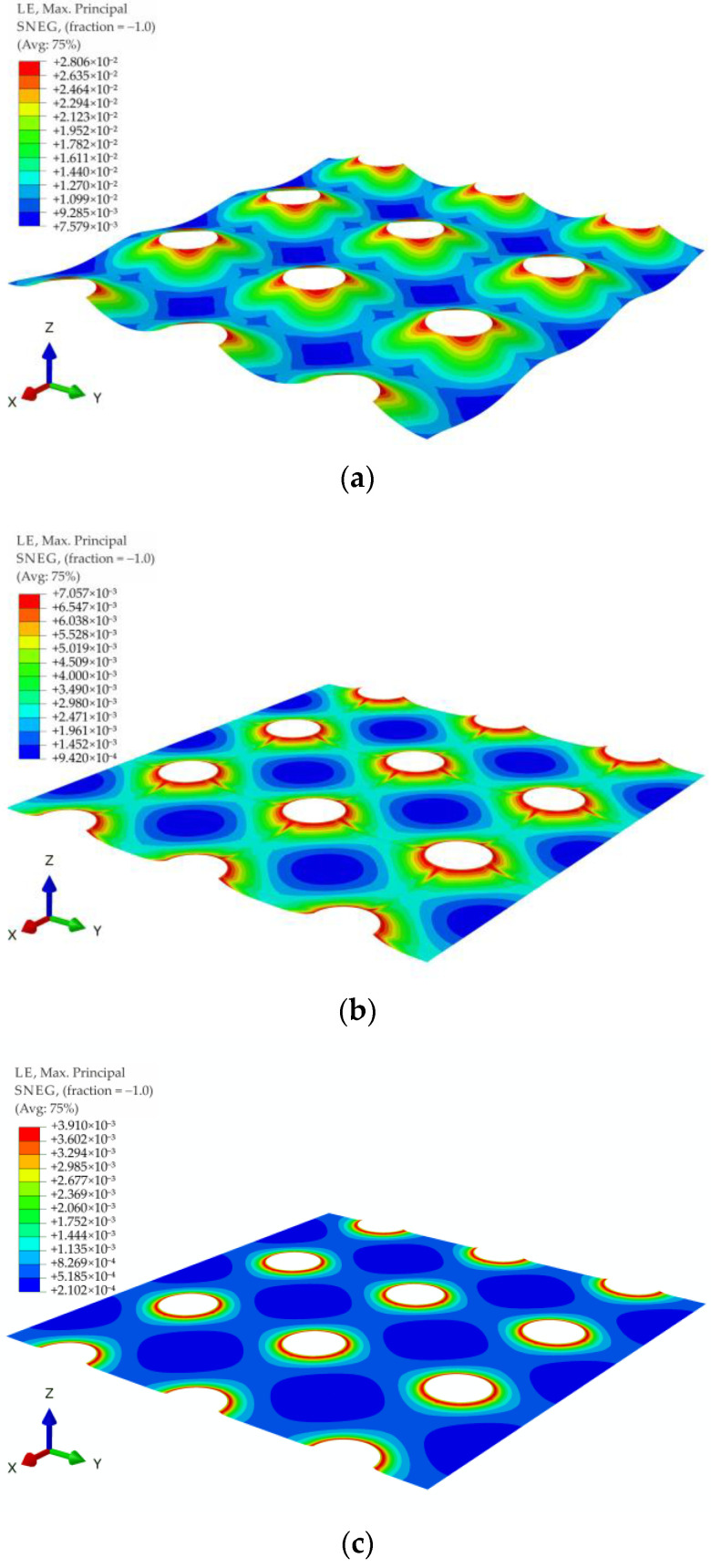
Deformation and maximum principal strain in reinforcement, variant B1: (**a**) k = 10 kN/m^3^; (**b**) k = 150 kN/m^3^; (**c**) k = 500 kN/m^3^.

**Figure 23:**
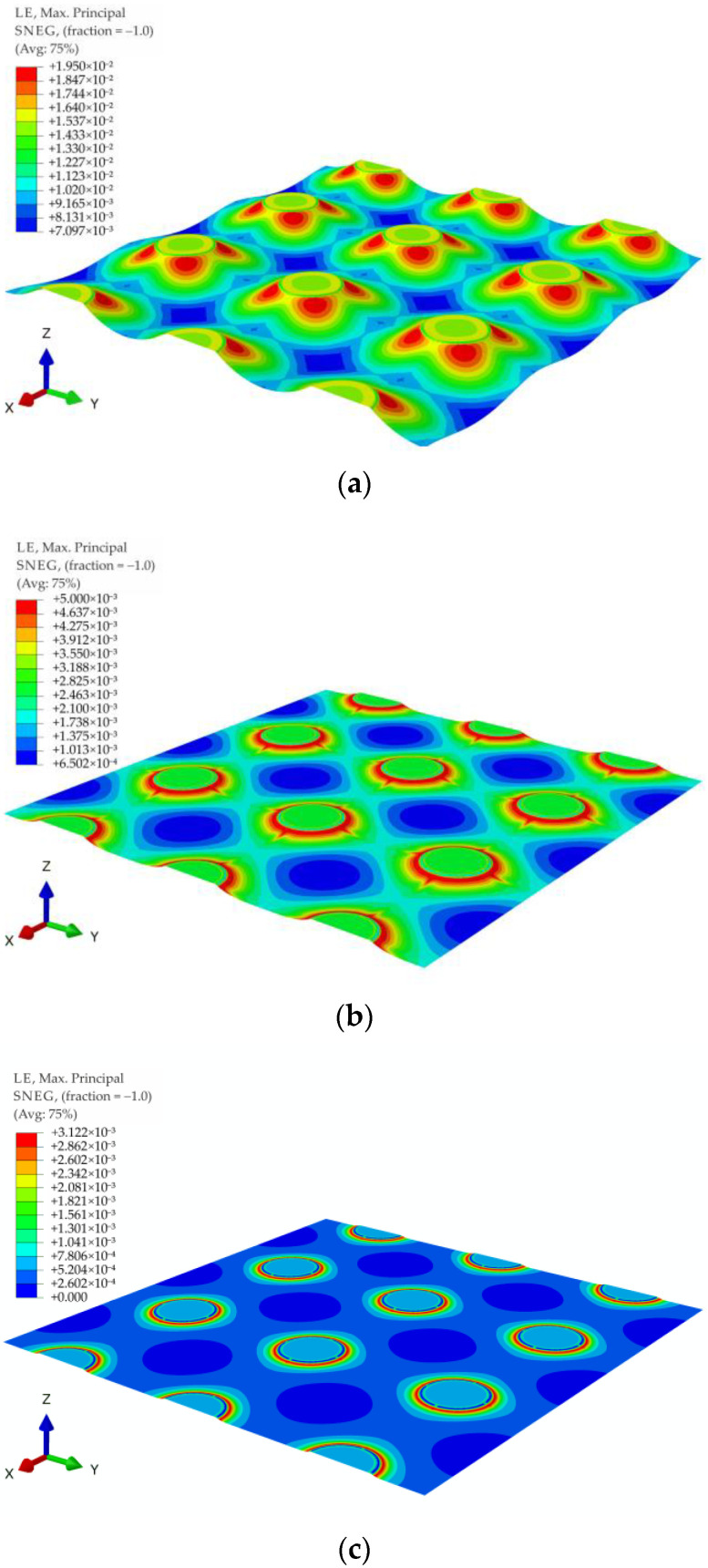
Deformation and maximum principal strain in reinforcement, variant B2: (**a**) k = 10 kN/m^3^; (**b**) k = 150 kN/m^3^; (**c**) k = 500 kN/m^3^.

Figure 22 and Figure 23 show the distribution of membrane maximum principal strain for the geometrical variants B1 and B2 for selected values of the subgrade reaction, respectively: k = 10 kN/m^3^, k = 150 kN/m^3^, k = 500 kN/m^3^. Also in the case of these variants, as in the case of the variants A1 and A2, for a small value of k, the cumulation of the largest strains at the column in the directions along the diagonals between the columns can be seen. However, when the cap above the column is modeled as circular, the strain along the circumference of the column even out earlier (the differences between the extremes decrease). Already for the ratio J/k = 10.77, an almost even distribution of the largest strains of the membrane around the pile cap can be observed (Figure 22b and Figure 23b).

**Figure 24:**
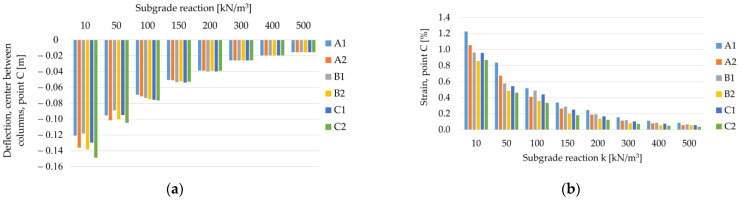
Membrane deflections and strain at point C depending on the subgrade reaction k and the geometric variant, J = 1615 kN/m: (**a**) deflection of the membrane at the point C; (**b**) strain at point C.

**Figure 25:**
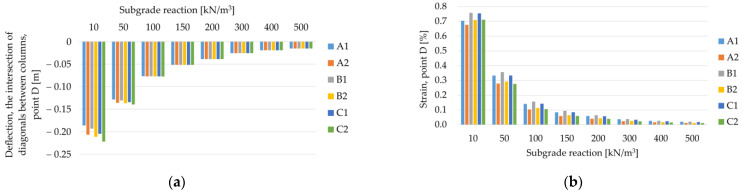
Membrane deflections and strain at point D depending on the subgrade reaction k and the geometric variant, J = 1615 kN/m: (**a**) deflection of the membrane at the point D; (**b**) strain at point D.

Comparing the maximum deflection of the membrane for different geometric variants and the range of subgrade reaction from 10 kN/m^3^ to 500 kN/m^3^ (Figure 24a and Figure 25a), it can be seen that for lower values of the subgrade reaction, the maximum deflection of the membrane at points C (in the middle of the span between the columns) and D (the intersection of diagonals between the columns, see Figure 3) differs depending on the analyzed geometric variant. On the other hand, for higher values of subgrade reactions, the values of maximum deflections are equal for all analyzed geometric variants. Deflections at point C are the same, regardless of the geometric variant for the ratio J/k = 5.38 (k = 300 kN/m^3^), and at point D for ratio J/k = 10.77 (k = 150 kN/m^3^). Interestingly, the strains at both points are different for different geometrical variants in the entire analyzed range of the subgrade reaction k (Figure 24b and Figure 25b). It is worth noting that Figure 20, Figure 21, Figure 22 and Figure 23 show solutions only for selected variants. This has not been noted in the analyzes presented by Zhang [38].

The obtained range of calculated strains indicates that it is necessary to pay more attention to adjusting the reinforcement stiffness to the occurring strains, which are smaller than 1.5%.

Further numerical analysis should be carried out for layered subsoil using soil model with stiffness varying with depth.

### 3.4. Effect of Load Distribution

In the analyzed FEM model, the load on the membrane between the columns was assumed to be uniform. In the case of LTP reinforcement of embankments on a soft soil improved with columns as a 2D task, there is a discussion about what membrane load distribution should be adopted to best map the real behavior of the reinforcement. This problem results from the fact that load distribution should model interaction from the layers above taking into account the arching effect in soil. The three most common distributions are the triangular distribution used in EBGEO [19] and the inverse triangular [26,42] and uniform [43,44,45] distribution used in CUR [27]. Translating this into a 3D problem, one can consider, apart from those usually used in numerical methods (cf. e.g., [38]), a pyramid shaped distribution corresponding to a triangular distribution in 2D, and an inverse pyramid distribution corresponding to an inverse triangular load distribution in 2D.

The distribution in the form of a pyramid in coordinate system like in Figure 3 is described by the function:

(1) $$f_{pyramid}=q_{p}\frac{y}{a}(1-\frac{x}{a}),$$

where:

$q_{p}$—the maximum value of the load at the top of the pyramid,
a—distance AB (compare Figure 3).

In turn, the inverse triangular distribution is described by the function:

(2) $$f_{inverse\;pyramid}=q_{ip}\frac{x}{a}(1-\frac{y}{a}),$$

where:

$q_{ip}$—the maximum value of the load at the edges of the column,
a—distance AB (compare Figure 3).

The load values $q_{p}$ and $q_{ip}$ have been calculated to ensure the equivalence of the total load applied to the membrane between the columns.

**Figure 26:**
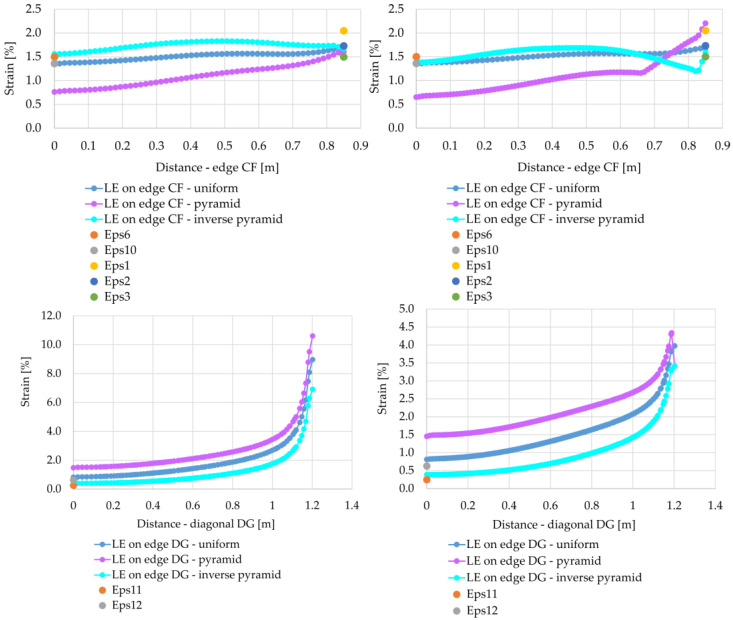

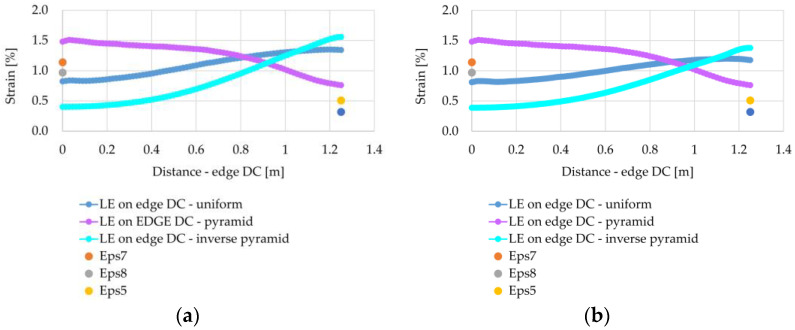
Comparison of the FEM calculation results for different load distributions with the results of strain measurements: (**a**) variant A1; (**b**) variant A2.

Comparison of the membrane strains for the load in the form of a pyramid and inverse pyramid for variants A1 and A2 with the results of measurements of reinforcement strain is shown in Figure 26. Analyzing the graphs presented in Figure 26, it is clearly visible that the adjustment of the membrane strains under the load of the inverse pyramid shows an even better fit to the results of measurements of deformations at the CF and DG edges, i.e., both in the direction between the columns and on the diagonal between the columns. This applies to both A1 and A2 variants. On the other hand, at the DC edge, the strains obtained for the pyramid shaped load distribution show a better match to the measurement results. Although FEM calculations gave higher values of strains in the case of the pyramid load distribution, but only in this case, the distribution of strains in the membrane at the DC edge corresponds to the measurements.

**Figure 27:**
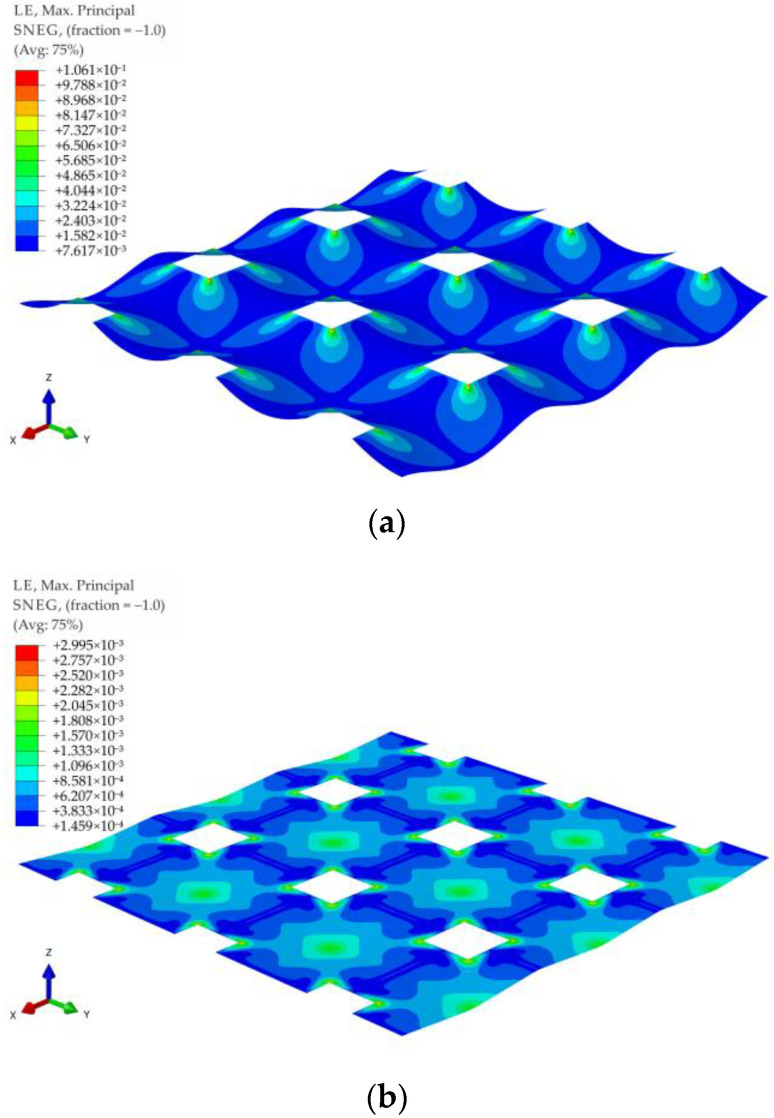
Deformation and maximum principal strain in reinforcement for pyramid shape load distribution, variant A1: (**a**) k = 0 kN/m^3^; (**b**) k = 500 kN/m^3^.

**Figure 28:**
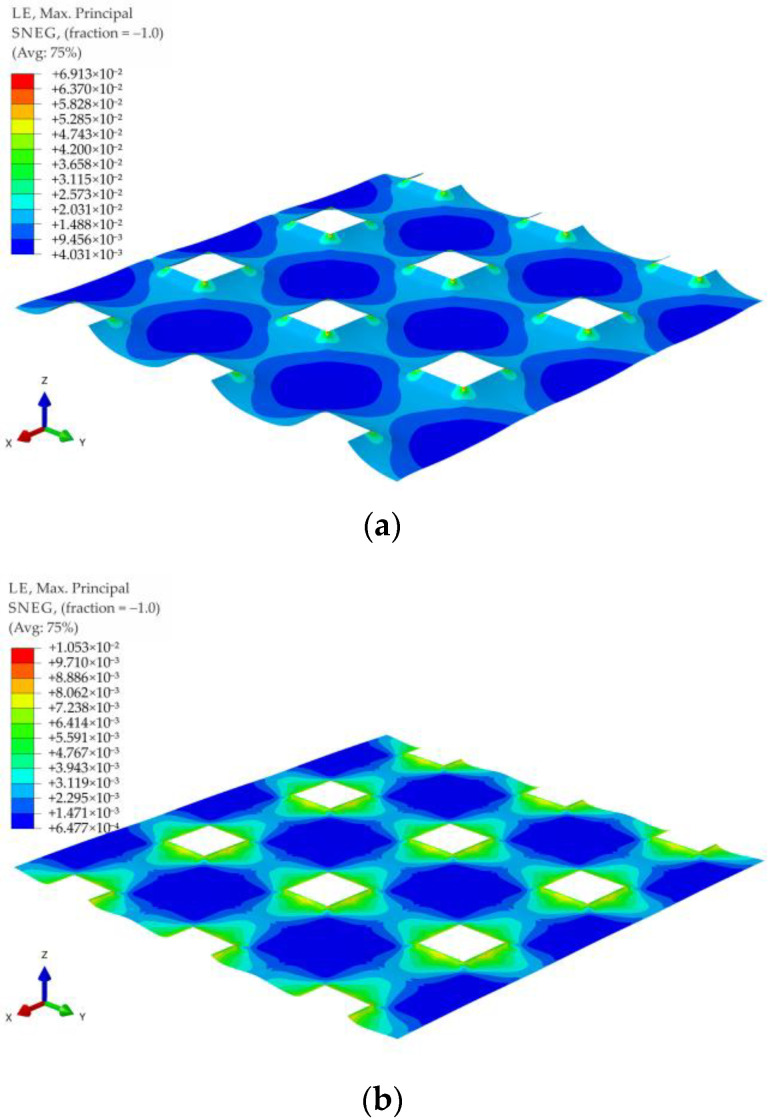
Deformation and maximum principal strain in reinforcement for inverse pyramid shape load distribution, variant A1: (**a**) k = 0 kN/m^3^; (**b**) k = 500 kN/m^3^.

**Figure 29:**
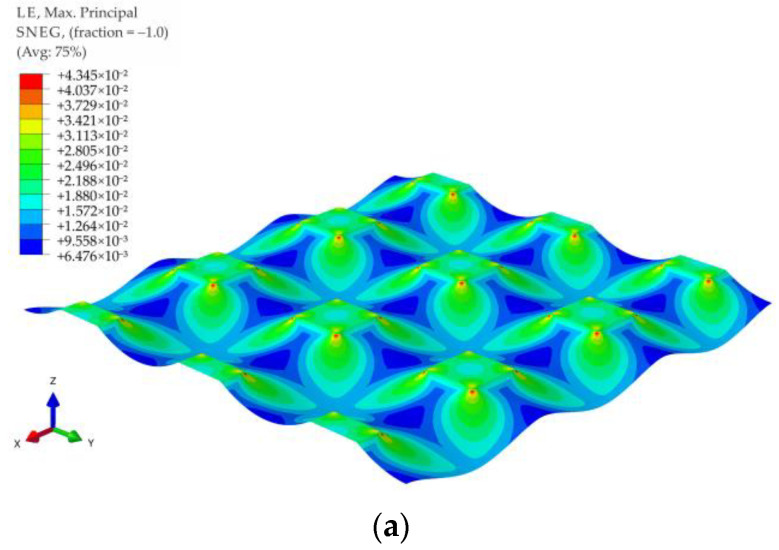

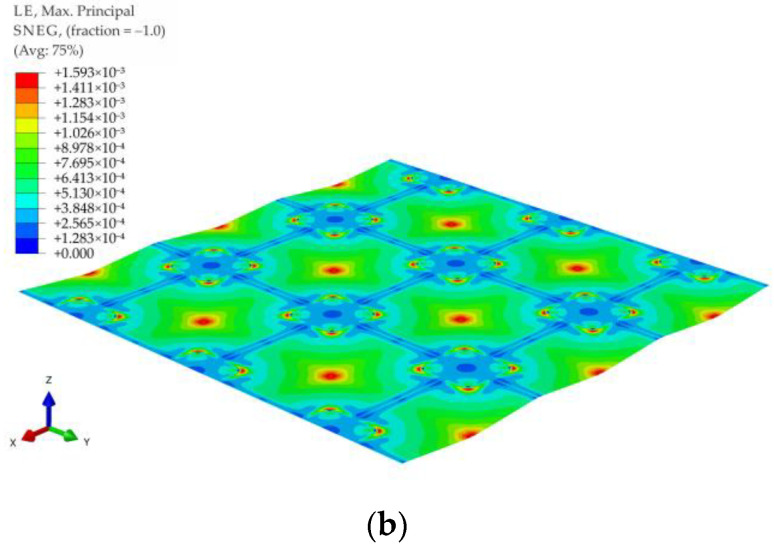
Deformation and maximum principal strain in reinforcement for pyramid shape load distribution, variant A2: (**a**) k = 0 kN/m^3^; (**b**) k = 500 kN/m^3^.

**Figure 30:**
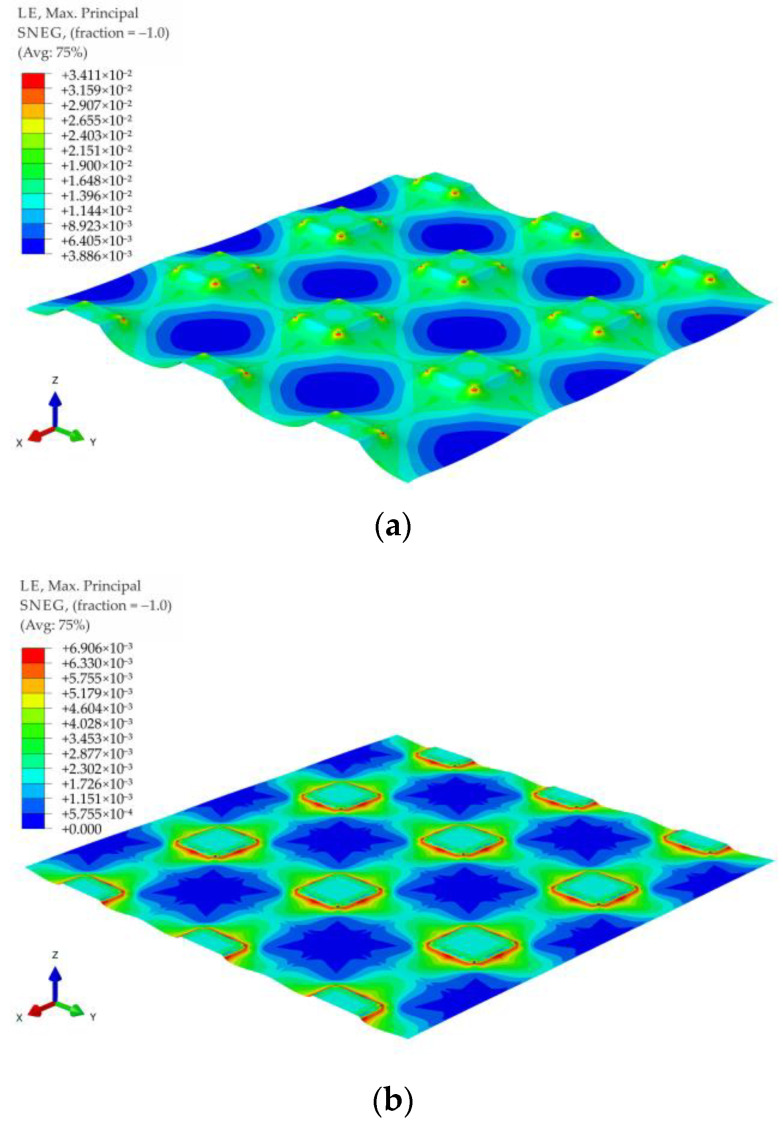
Deformation and maximum principal strain in reinforcement for inverse pyramid shape load distribution, variant A2: (**a**) k = 0 kN/m^3^; (**b**) k = 500 kN/m^3^.

**Figure 31:**
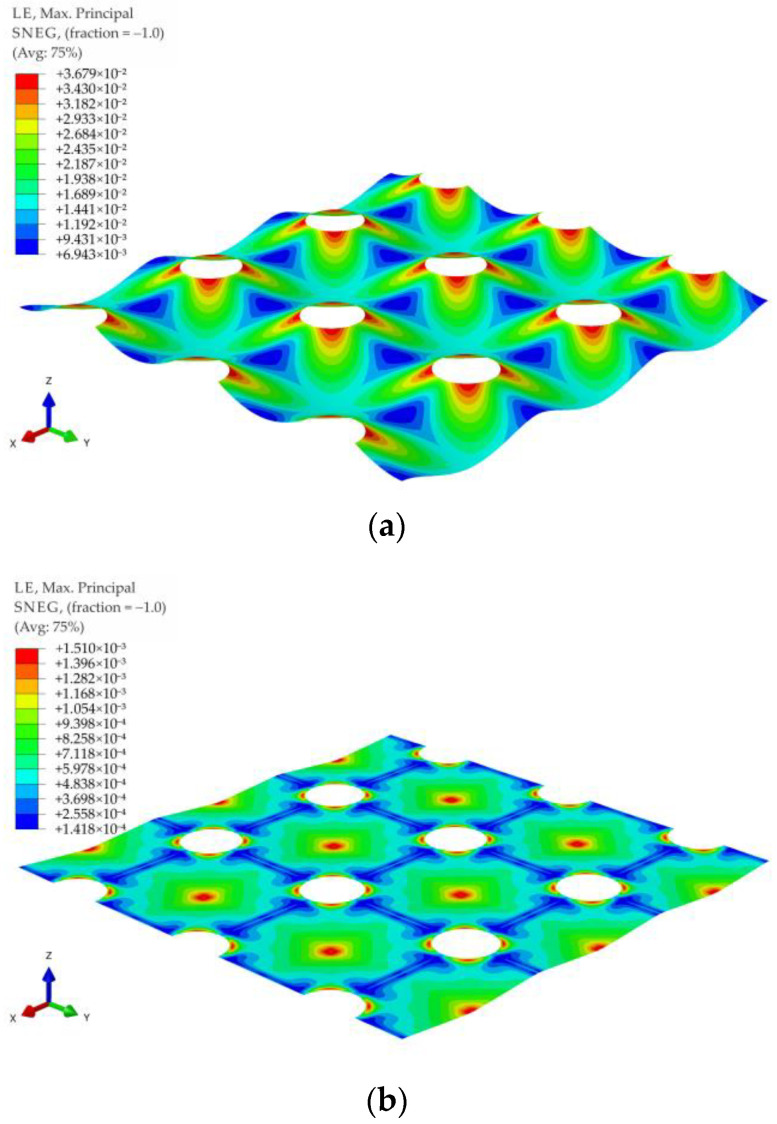
Deformation and maximum principal strain in reinforcement for pyramid shape load distribution, variant B1: (**a**) k = 0 kN/m^3^; (**b**) k = 500 kN/m^3^.

**Figure 32:**
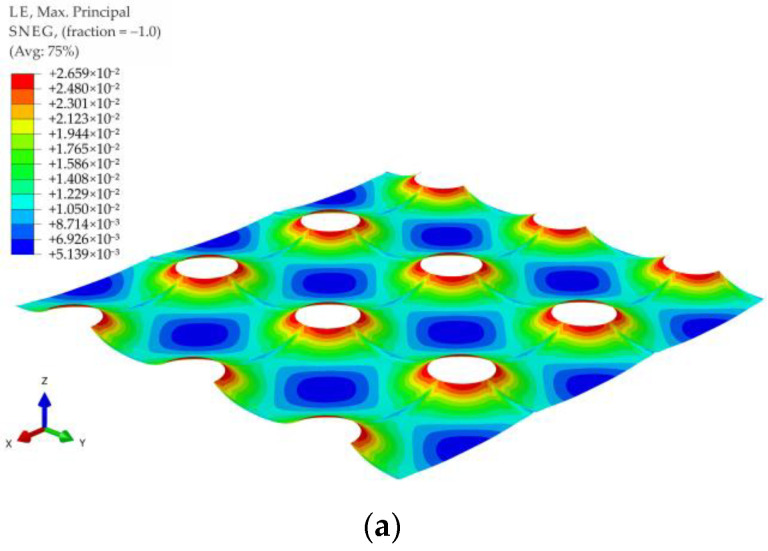

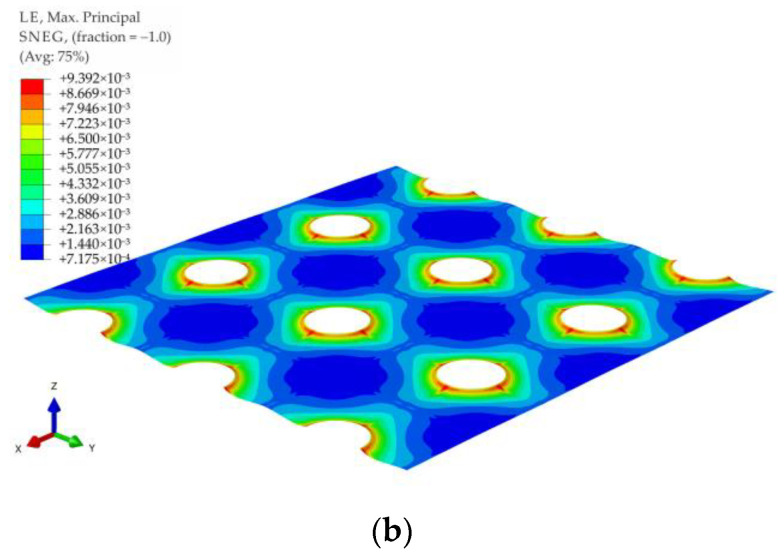
Deformation and maximum principal strain in reinforcement for inverse pyramid shape load distribution, variant B1: (**a**) k = 0 kN/m^3^; (**b**) k = 500 kN/m^3^.

**Figure 33:**
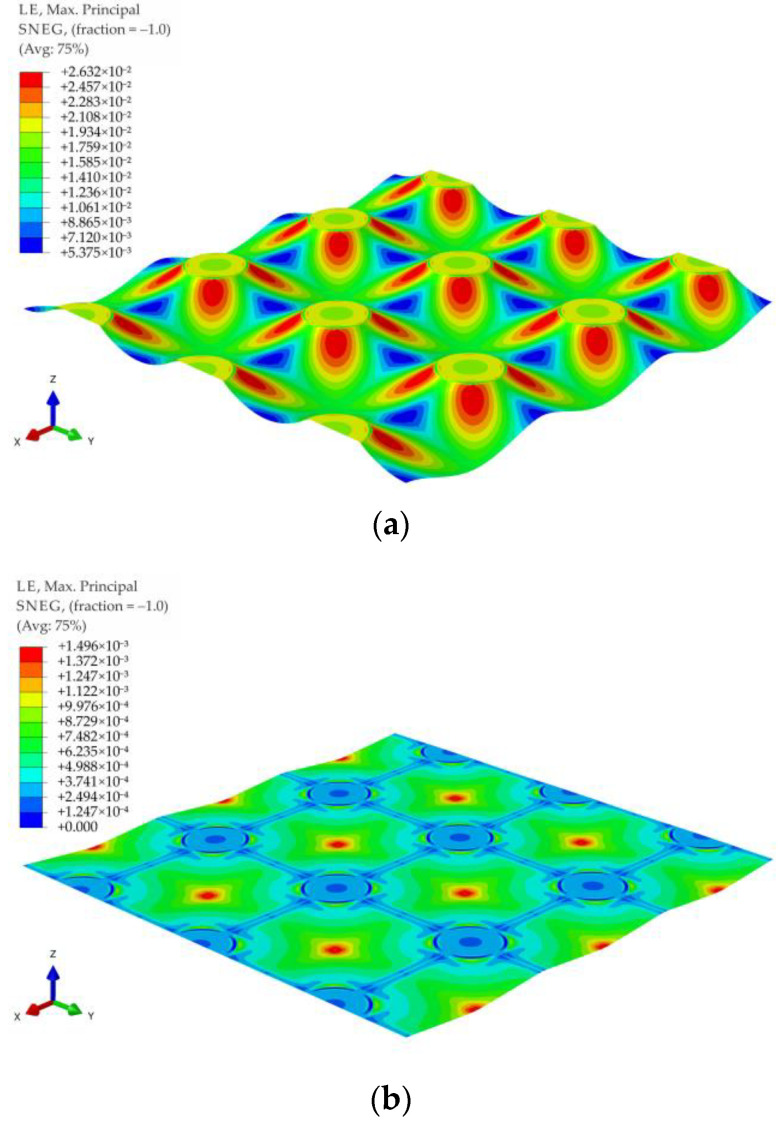
Deformation and maximum principal strain in reinforcement for pyramid shape load distribution, variant B2: (**a**) k = 0 kN/m^3^; (**b**) k = 500 kN/m^3^.

**Figure 34:**
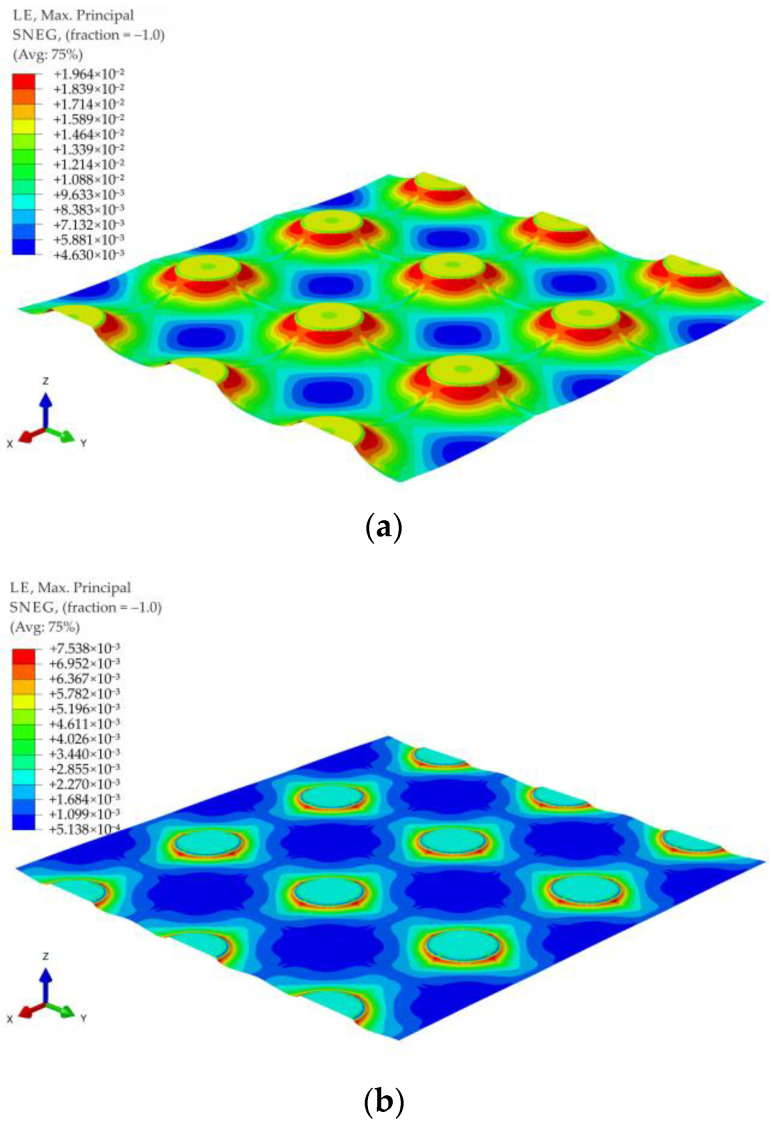
Deformation and maximum principal strain in reinforcement for inverse pyramid shape load distribution, variant B2: (**a**) k = 0 kN/m^3^; (**b**) k = 500 kN/m^3^.

The maximum principal strains contour graphs for the membrane modeling LTP reinforcement in the case of no soil support (k = 0) and significant soil support (k = 500 kPa) for the variant A1 and load distribution in the form of a pyramid are shown in Figure 27, and for the variant A1 and load distribution in the form of an inverse pyramid are shown in Figure 28. Analogical graphs for the variants A2, B1, and B2 for the load distribution in the form of a pyramid and inverse pyramid are shown in Figure 29, Figure 30, Figure 31, Figure 32, Figure 33 and Figure 34.

When analyzing the maximum principal strains presented in the contour charts, it can be stated that the load distribution radically changes the position of the extreme values in the case of significant values of subgrade reaction, cf. e.g., Figure 29b and Figure 32b, as well as Figure 33b and Figure 34b. In the case of no soil support (or a very low value of subgrade reaction coefficient), the extremes are located around the caps of the columns, and only the distribution around caps’ circumference changes.

Summarizing, as it is difficult to clearly indicate the membrane load distribution in 2D conditions, which in all situations allowed to reflect the actual behavior of the LTP reinforcement [46], in the case of 3D, the answer is not unequivocal. Further research is needed to look for universal models describing the behavior of embankments on a soft soil improved with columns with reinforced load transfer platform.

## 4. Conclusions

The studies allowed for the analysis of the behavior of the membrane modeling the reinforcement of the load transfer platform (LTP) of the embankments on a soft ground improved with columns. Based on the research, the following conclusions can be made:

1. In the case of the arrangement of columns in a square and the absence of soft soil (k = 0 kN/m^3^), the greatest strains of the reinforcement are located in the direction of diagonals between the columns. This happens regardless of the shape of the column/cap (square, round) and the boundary conditions used. Interestingly, the greatest strains are not always located at the edge of the cap, but at some distance from the cap.
2. The stiffness value of a soft soil significantly affects both the value and distribution of strains in the membrane. As the soft soil subgrade reaction coefficient k increases gradually, the highest strain begins to localize around the cap. In the case of round-shaped caps, the leveling of strains around the cap occurs much faster than in the case of square caps. In the case of square caps (variant A2) for the ratio J/k = 3.23, the uniform distribution of the biggest membrane strain around the column can be seen, while in the case of round caps, an almost even distribution of the largest strains of the membrane around the pile cap can be observed for the ratio J/k = 10.77.
3. The value of the Poisson’s ratio adopted for the membrane material has an impact on the strain distribution in the reinforcement modeling membrane. This influence is visible both in the case of the strain values at the edge of the column and in the middle of the span between the columns, as well as the location of the maximum strains in relation to the edge of the column.
4. In numerical calculations, the manner of implementing the boundary conditions has a significant impact on the values and distributions of individual quantities. Thus, in practical applications, the choice of proper boundary conditions in particular cases is an essential thing.
5. The influence of geometry on the deflection values is visible when the soft soil between the columns is characterized by no or low stiffness values. With the increase of the soft soil stiffness (reduction of the ratio of the reinforcement stiffness to the subgrade reaction coefficient), the influence of the geometric variant on the maximum deflection of the membrane is no longer visible. However, the values of strains in the points of maximum deflection remained different, depending on the geometric variant in the entire range of the analyzed ratios of reinforcement stiffness to soft soil stiffness (subgrade reaction k).
6. The use of round caps prevents stress concentration in the corners and significant strain values of the reinforcement in these places.
7. Proposed load distributions in 3D conditions in the form of a pyramid and an inverse pyramid are original when applied for LTP modelling. The strain distributions and maximum strain values are verified by comparison with experimental data. Both proposed distributions properly model the shape functions in cross-sections CF and DG, but only pyramid shape distribution allows for proper modelling of the strain function in cross-section DC. On the other hand, the values obtained with assumption of the load equivalence are not equal to those observed in the experiment, so the natural consequence may be for example neglecting this assumption and looking for appropriate scaling factors.

## Figures and Tables

**Table 1:** Results of geosynthetic strain measurements.

At the Edge of the Cap			Between Columns (Caps)							
$\varepsilon_{1}$	$\varepsilon_{2}$	$\varepsilon_{3}$	$\varepsilon_{5}$	$\varepsilon_{6}$	$\varepsilon_{7}$	$\varepsilon_{8}$	$\varepsilon_{9}$	$\varepsilon_{10}$	$\varepsilon_{11}$	$\varepsilon_{12}$
2.05%	1.73%	1.50%	0.51%	1.50%	1.14%	0.97%	0.32%	1.36%	0.25%	0.63%

**Table 2:** Parameters used in the analysis related to the presented experiment.

Axial Spacing of Columns	Head Width	Embankment Height	Volumetric Weight of the Embankment	Internal Friction Angle	Stiffness of the Reinforcement
2.5 m	0.8 m	1.25 m	18 kN/m^3^	60° *	1615 kN/m

* the value of the internal friction angle was determined based on the actual internal friction angle and the cohesion of the fill material.

**Table 3:** Summary of the number of finite elements S3 and S4R depending on the analyzed geometric variant.

Variant	A1	A2	B1	B2	C1	C2
Number of elements	7583	18,712	7563	19,139	7708	19,131

## Data Availability

No new experimental data were created in this study. Data sharing is not applicable to this article. The calculation data presented in this study is available at the request of the respective author.
